# Illumination of a Vision—How Arthur Rimbaud Will Give Us Motivation to Find New Input into Bladder Cancer Biomarker Research

**DOI:** 10.3390/ijms18112463

**Published:** 2017-11-19

**Authors:** Thorsten H. Ecke, Thomas Otto

**Affiliations:** 1 Department of Urology, HELIOS Hospital Bad Saarow, 15526 Bad Saarow, Germany; 2 Department of Urology, Lukaskrankenhaus Neuss, 41464 Neuss, Germany; thomas_otto@lukasneuss.de

Bladder cancer (BC) accounts for approximately 430,000 new cases and 165,000 deaths each year worldwide [1,2]. Around 30% of bladder cancer patients suffered from muscle- invasive bladder cancer (MIBC) at the time of first diagnosis [3]. The incidence of urinary bladder cancer has increased in the last decades. Bladder cancer has a high rate of recurrence and a significant number of non-invasive tumours will progress to muscle-invasive disease. Due to the heterogeneity of the tumour, new markers for tumour progression are clearly needed as clinical parameters, such as tumour grade and stage are not accurate in predicting the biological behaviour and thus guiding the choice of treatment, especially in high risk cases [4,5,6].

It seems that urinary-based assays could detect the presence of bladder cancer, because the malignancy is in direct contact with urine. Malignant cells are shed into the urine, and it is likely that urine will contain carcinogens producing the malignancy. But the illusion or vision—again Rimbaud—that one single molecular marker can detect all kinds of bladder cancer accurately is probably not correct [7]. Clinical evidence and molecular studies suggest that there are two pathways in human bladder carcinogenesis: the pTa pathway and the carcinoma in situ (CIS) pathway [8]. pTa tumours are mostly low-grade and often recur, but rarely progress to lamina propria-invasive (pT1) and muscle-invasive tumours (pT2–T4), whereas CIS are always high-grade and are thought to be the most common precursor of invasive tumours. Interestingly markers detecting tumours at low-grade pathway as FGFR3 for example have only a 7% overlapping mutation rate comparing to markers detecting tumours at high-grade pathway as TP53 [9,10]. A urinary-based assay that can diagnose bladder cancer whilst confined to the urothelium or carcinoma in situ could fulfil the criterion to differ between both. This model has also been confirmed by other publications in the past [9,11,12].

Not focus on one marker is the goal, we need to study a combination of several different tumor markers to guide the interval between cystoscopies, and to direct biopsy of clinically meaningful “occult” disease that could not be detected by regular histopathological reports [13]. New markers should be based on their characteristics as well as the particular risk profile of the studied patients. This could lead to greater sensitivity than either marker alone, but worsens overall specificity.

Some promising bladder cancer markers have even a better accuracy than prostate-specific antigen has for prostate cancer screening [14,15]. It will depend by the willingness of physicians and patients whether one of the bladder tumor markers will find more influence in the clinical treatment and the changing of the diagnosis of bladder cancer in future.

Further determination of recurrence and progression marker will contribute to establish better treatments for the individual patient. Molecular staging of urological tumors will allow selecting cases that will require systemic treatment [16,17]. Regarding new therapies, it is also important to know more about cancer progression pathways which allows the evaluation of medical therapies against these specific tumor targets. In order to obtain such objectives, it is necessary to integrate basic and clinical research teams. Such teams would require integration of clinical follow-up information of cancer patients with optimal tumor and serum banks. However, the most important task is to integrate under the same objectives basic and clinical research.

On the other side it should be a need in all further studies about bladder cancer markers to have a very clear classification of the studies into phases I to IV; this had already been recommended by Lokeshwar et al. in 2005 [18]. In that meaning phase I are always feasibility studies showing development and evaluation of clinical prevalence for assays, phase II are including all evaluation studies for clinical utility, phase III are confirmation studies, and phase IV are application studies for validation and technology transfer. Having this in mind we will account these recommendations in the presented studies in our special issue.

Clinical needs in the uro-oncology are related to diagnosis, prognosis and treatment. Uro-oncology is diverse since genitourinary tumors differ histologically in their origin and various clinical behaviour [19].

Another important fact is that bladder cancer is one of the most expensive malignancies in the Western countries, the cost of a bladder cancer patient from diagnosis to death was calculated between $96,000 and $187,000 in 2001 [20,21]. Therefore bladder cancer markers are needed in future to reduce cost intensive and also painful examinations like cystoscopies, and define risk groups to know in advance which treatment is the best for the patient.

This special issue has been introduced with the aim of offering the possibility to publish new research results from old and new pioneers in the field of bladder cancer basic research. While editing this special issue we have learned that an enormous enthusiasm is necessary to go on in bladder cancer research. In our eyes bladder cancer is on one hand a very heterogenous malignancy that’s why it makes so difficult to focus on the one and only bladder cancer marker in bladder cancer diagnostic and follow-up. On the other hand bladder cancer has a high importance to find prognostic and predictive factors due to its high incidence and its enormous costs as one of the most expensive malignancies in the world. Finding and development of new bladder cancer markers is still a very dynamic field. Because of the mass of all these markers it is impossible to report all. This special issue is trying to highlight the role of bladder cancer markers in diagnosis and the most important biomarkers studied and reported recently. In this special issue a highlight to some of the most important markers was made. Further determination of recurrence and progression marker will contribute to establish better treatments for the individual patient. Molecular staging of urological tumors will allow selecting cases that will require systemic treatment. It is necessary and important to integrate under the same objectives basic and clinical research.

If scientists/medicines and artists come in contact, the mixture will bring new energy into each of them. This will happen very often, when scientists/medicines love art—what all of them should do. On the other side it could happen if artists come in contact with medicines—what mostly is a need, not a will. Let us find out, what are parallels between bladder cancer research and French poetry: Arthur Rimbaud (Figure 1) was a pioneer in modern poetry and showed a clear way for all poets following him. Pioneers in medicine are also showing a clear way for their followers, especially diagnosis for bladder cancer is a very progressive field in medicine, though there are still two very old diagnostic standards—haematuria and cystoscopy. All what follows has to be measured with that standards. Due to different pathways in bladder cancer and the heterogeneity of this disease there are many possibilities to go into its mechanisms. White is not only white—black is not only black—Arthur Rimbaud would say “L’Étoile a pleuré rose…” (English: “The star wept rose…”). At the end he suffers from a malignancy. At the end science could perform new ways (Figure 2: Author T.E. walking on Rimbaud’s routes close to Charleville in France) to help patients with instruments for early diagnostics and with predictive and prognostic markers finding new and personalized strategies for therapy.

The editors thank all submitting authors for their efforts and time spent for each manuscript. The lead editor would like to thank all editors for the time spent in reviewing, assigning reviews, and commenting on submitted manuscripts. As editorial team, we hope that this special issue will prove useful to research work in bladder cancer in future. Hopefully many researchers will use any kind of art to improve their professional success to ameliorate diagnostics and therapy in bladder cancer.

## Figures and Tables

**Figure 1:**
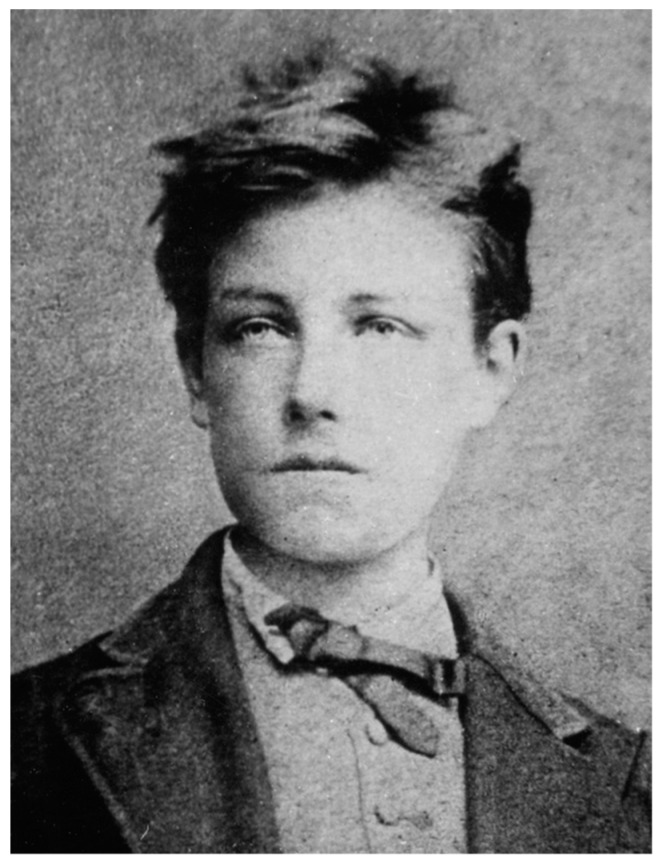
Arthur Rimbaud (1872)—photography by Étienne Carjat.

**Figure 2:**
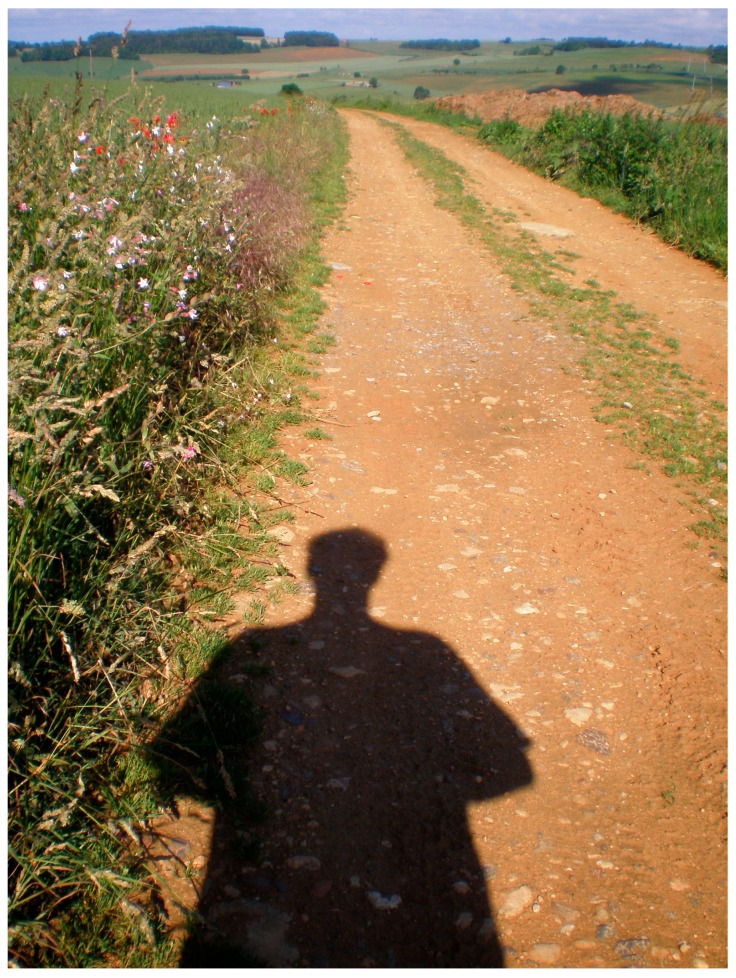
Author Thorsten H. Ecke walking on Rimbaud’s routes close to Charleville in France.
